# Experiences and Coping Strategies of Emergency Department Nurses Regarding Unethical Pro‐Organizational Behavior: A Descriptive Qualitative Study

**DOI:** 10.1155/jonm/4212336

**Published:** 2026-10-19

**Authors:** Juan Qiao, Junjie Ma, Huiling Feng, Shanshan Zhang, Lin Wang, Minghui Zhou, Hanfei Cui, Xuebing Jing

**Affiliations:** ^1^ Nursing Department Zibo Central Hospital Zibo China zbzxyy.com; ^2^ Emergency Department Zibo Central Hospital Zibo China zbzxyy.com; ^3^ Integrated Traditional Chinese and Western Medicine Orthopedic Department Zibo Central Hospital Zibo China zbzxyy.com; ^4^ Clinical Trial Center Zibo Central Hospital Zibo China zbzxyy.com

**Keywords:** descriptive qualitative study, emergency department nurse, nursing ethics, unethical pro-organizational behavior

## Abstract

**Background:**

Emergency nurses’ unethical pro‐organizational behaviors (UPBs) exert detrimental effects on individual well‐being, professional performance, and clinical workplace atmosphere; nevertheless, qualitative evidence systematically exploring the underlying mechanisms and coping strategies of such behaviors in the Chinese nursing context remains scarce.

**Aim:**

This study aimed to qualitatively explore the sources, perceived impacts, and coping strategies of UPBs among emergency department nurses in China.

**Design:**

Descriptive qualitative study.

**Methods:**

This study was conducted in the emergency department nurses of five hospitals in China between February and March 2026. A total of 21 registered nurses aged ≥ 18 years who were currently in practice were recruited using purposive sampling. Data were collected using semistructured interviews until thematic saturation was achieved. Data analysis was performed using qualitative content analysis.

**Results:**

Three main themes emerged—sources, impacts, and coping—highlighting that UPBs arose from high‐pressure environments and distorted loyalty, led to moral distress and normalization, and was managed with adaptive and self‐rationalizing strategies.

**Conclusions:**

UPBs among emergency nurses constitute a vicious cycle fueled by workplace stressors, implicit cues, and coping strategies, which profoundly affects individual well‐being and organizational ethics. These findings suggest that source‐oriented humanistic interventions, including environmental optimization, ethical support, competence enhancement, and cultural reshaping, are essential for practice.

**Implications for Nursing Management:**

Nursing managers should target systemic and cultural drivers of UPBs, clarify ambiguous expectations, establish ethical support (e.g., confidential reporting and debriefings), model integrity‐based leadership, and decouple loyalty from unethical compliance. These actions are designed to alleviate ethical distress, reduce rule‐bending, and break the vicious cycle in emergency settings.

**Reporting Method:**

This study was reported in accordance with the Consolidated Criteria for Reporting Qualitative Research guidelines.

**Patient or Public Contribution:**

No patient or public contributions.

## 1. Introduction

Ethical issues have increasingly become critical factors influencing the quality of care and patient safety in modern healthcare institutions. Unethical pro‐organizational behaviors (UPBs) have emerged as covert, yet they pose severe challenges in nursing practice [1]. UPBs refer to actions taken by employees for organizational benefits that violate core social values, moral norms, or appropriate conduct standards, such as concealing negative information or exaggerating service quality [2]. These behaviors exhibit two key attributes: “pro‐organizational nature,” implying that they are intended to serve organizational or member interests, and “unethical nature,” meaning they contravene universal moral or legal standards. Driven by perceived organizational pressure, context‐specific pro‐organizational unethical behaviors may bring short‐term benefits to the organization and help individuals achieve their goals within it. However, the existing literature suggests that such behaviors are potentially associated with long‐term declines in organizational governance quality and may undermine sustainable institutional development [3, 4].

Scholars have proposed multifaceted explanations for the mechanisms underlying the formation of organizational UPBs. Early research suggested that such behavior is primarily shaped by organizationally proximate motivations and identified factors such as organizational identity [2], emotional commitment to the organization, positive reciprocity beliefs, and perceived organizational support as key correlates of employees’ engagement in UPBs [5–7]. However, some scholars argue that employees may perceive organizationally proximate unethical behavior as a “shortcut” to meet organizational demands, such as resorting to inappropriate means under high‐pressure performance targets [8]. Research has shown that overqualified employees are more likely to engage in UPBs to maintain high self‐evaluations and sustain this “inflated” positive self‐image [9]. When employees seek to align themselves with leadership expectations and meet the baseline targets set by superiors, they may be inclined to employ unethical means to achieve these goals [10]. These perspectives collectively suggest that organizational UPBs arise not from a single motivational source but from a confluence of genuine pro‐organizational sentiments, pragmatic responses to performance pressures, and individual self‐evaluation concerns, particularly when employees perceive unethical actions as viable means of fulfilling both organizational and personal goals.

Furthermore, healthcare systems across various countries encounter challenges in nurses’ ethical decision‐making; however, the specific manifestations and response strategies vary. For instance, healthcare management in certain countries places stronger emphasis on individual accountability and a culture of transparency [11, 12]. In contrast, professional practice in Chinese healthcare settings is embedded in a localized social context shaped by Confucian relational ethics, which prioritizes interpersonal harmony, hierarchical professional norms, and collective team interests. Within the local institutional and workplace context, situational collectivist norms and hierarchical professional obedience may contextually encourage clinical nurses to prioritize relational and collective workplace harmony over individual ethical autonomy, occasionally leading to pragmatic ethical compromises to accommodate institutional operational demands [13]. Notably, these cultural and relational factors are not fixed national deterministic traits but dynamic contextual variables that interact with institutional environments. Institutional mechanisms supporting nurses’ independent ethical voice appear relatively limited, leaving frontline nurses hesitant to voice ethical concerns when confronting clinical dilemmas. Additionally, localized interpersonal relational norms (guanxi) and face‐saving considerations (mianzi), which are prevalent in Chinese workplace contexts, interact with institutional resource allocation rules and daily clinical interactions to complicate ethical decision‐making [14]. Unlike prior studies that frame these cultural elements as static, inherent national characteristics, the present study conceptualizes cultural influences as flexible, context‐dependent workplace dynamics that intersect with organizational constraints to shape frontline nursing practice. These relational and hierarchical contextual features create unique situational ethical challenges that differ from individualism‐oriented Western healthcare environments.

In routine clinical work, openly challenging senior physicians or nurse leaders may be interpreted as interpersonal disrespect, potentially disrupting long‐term professional partnerships and future collaborative workflows [15, 16]. Against this contextual backdrop, nurses tend to resolve ethical concerns through informal private communication rather than formal institutional channels, a contextually adaptive workplace strategy [13]. While such relational coping strategies help maintain superficial operational harmony and team stability, they may obscure formal ethical accountability boundaries. When ethical conflicts are resolved through informal relational networks rather than standardized institutional procedures, systematic mechanisms for recording, tracking, and addressing ethical risks are often absent, leaving underlying ethical dilemmas unaddressed. In this sense, localized guanxi and mianzi workplace norms serve as situational contextual influences that constrain nurses’ ethical autonomy and complicate principle‐based ethical practice in the sampled healthcare context, rather than representing fixed national cultural determinants.

To establish a clear conceptual foundation for investigating UPBs among emergency nurses, it is essential to delineate the boundary conditions of this construct. According to prior theoretical work [2], UPB is defined by three criteria: (1) it must be an intentional act, not one performed by mistake, error, or unconsciousness; (2) it is judged based on the actor’s starting point (i.e., the intention to benefit the organization or its members) rather than the actual outcome; and (3) behaviors driven primarily by self‐interest, rather than organizational or member interests, do not constitute UPB. These criteria conceptually distinguish UPB from several adjacent but distinct phenomena commonly observed in nursing practice. Unlike clinical errors, which are unintentional and often system‐driven, UPB involves deliberate ethical transgressions. Unlike organizational disobedience, which entails active resistance to organizational rules or authority, UPB is performed with the explicit aim of supporting organizational interests. Unlike professional silence, which reflects passive withholding of voice due to fear or power asymmetry, UPB entails active, voluntary actions. Unlike moral distress, which arises from knowing the right course of action but being constrained from taking it, UPB actors actively choose unethical means to achieve perceived organizational goods. Unlike moral disengagement, where individuals rationalize misconduct to avoid self‐condemnation, UPB actors are typically aware of the unethical nature of their actions but justify them through prosocial organizational motives. Finally, unlike clinical rule‐bending or workarounds, which are often pragmatic responses to inefficient systems or resource shortages, UPB is specifically driven by a desire to protect or promote organizational interests, even at the expense of ethical standards. Current research on nurse behaviors has dealt chiefly with workplace bullying [4], toxic leadership behaviors [5], professional misconduct [6, 7], disruptive behavior [5], medical error reporting [8], counterproductive work behaviors [9], organizational citizenship behavior [10], and proactive work behavior [11]. The literature indicates that nurses’ unethical behavior is typically self‐interested, potentially harming the organization and its members, which fundamentally differs from UPBs. Clarifying these conceptual boundaries is particularly critical in emergency department settings, where fast‐paced decisions and systemic pressures may blur the line between intentional unethical pro‐organizational actions and routine clinical workarounds.

As the primary clinical frontier for life‐threatening and time‐sensitive conditions, emergency department nurses are characterized by rapidly evolving patient presentations that frequently raise intricate ethical issues, including life‐sustaining interventions, informed consent, and resource allocation [17]. Nurses in this high‐acuity environment must make prompt decisions that are clinically proficient and ethically defensible, making them particularly susceptible to moral distress and occupational strain [18]. Moreover, the perceived ethical support and self‐assessed ethical competence of emergency nurses directly affect their job satisfaction and burnout levels [19]. However, presently, the performance‐oriented management system of medical institutions, imbalanced allocation of medical resources, and other problems are likely to cause UPBs among medical staff [20]. Therefore, a systematic investigation of UPBs among emergency nurses is essential for enhancing nursing quality, protecting nurses’ well‐being, and maintaining a stable emergency care workforce.

Existing research includes qualitative studies examining nurses’ perceptions of UPBs in general wards and various quantitative studies investigating influencing factors (e.g., ethical leadership and organizational support) [5, 21]. A review of the existing literature suggests three interconnected areas that remain underexplored. First, methodologically, while quantitative studies have identified statistical associations between various factors (e.g., ethical leadership and organizational support) and UPBs, they cannot illuminate the lived experiences, subjective meanings, and decision‐making processes underlying ED nurses’ UPB. A recent qualitative study explored nurses’ general perceptions of UPB in hospital settings in Chengdu, China [4]; however, it was conducted in general wards rather than in the unique context of emergency department nurses. Second, contextually, the ED represents a distinct care environment characterized by extreme time pressure, information uncertainty, task interruptions, and life‐and‐death decisions. These factors fundamentally alter the ethical decision‐making landscape compared with that of general wards; however, no study has specifically examined how these emergency department–specific characteristics shape nurses’ engagement in UPBs. Third, culturally, China’s healthcare system operates within a cultural framework of collectivism, hierarchical obedience, and relational ethics (“guanxi”/“mianzi”) that fundamentally differs from that of Western healthcare contexts. Existing UPBs research has been predominantly conducted in Western organizational settings, and the findings may not be directly transferable to the Chinese context. A review of the literature across PubMed, Web of Science, China National Knowledge Infrastructure, and CINAHL databases reveal that existing qualitative inquiries have largely examined nurses’ UPBs from a general departmental perspective. Notably, Li et al. (2025) conducted a descriptive qualitative study in Chengdu using the Theory of Planned Behavior, exploring nurses’ perceptions and experiences of UPBs across multiple hospital settings [4]. However, their sample was not restricted to the emergency department. This limitation hinders a comprehensive understanding of nurses’ authentic experiences in emergency settings and the underlying mechanisms linking situational factors to individual behaviors, thereby restricting the development of effective intervention strategies.

Social cognitive theory (SCT), proposed by Bandura, is built upon the core architecture of triadic reciprocal determinism, which posits that human activity arises from the dynamic interaction of three factors: organizational environmental factors, personal cognitive appraisals, and behavioral responses. The theory asserts that these three elements are not linked by unidirectional causal relationships but rather form an intricate, mutually influential, and constantly evolving system [22]. Specifically, the environment shapes individuals’ psychological states and behaviors, while individuals’ actions in turn modify the environment. Individuals are not passive respondents to their surroundings; their internal psychological attributes actively guide their behavioral choices. Behavior, meanwhile, is neither simply determined by the person nor by the environment; it emerges as the concrete expression of their interaction, and the outcomes of behavior feed back to both the individual and the environment. Together, these three components constitute a complex and dynamic whole. Therefore, to understand an individual’s behavior, one must simultaneously examine both the external environmental factors and the internal psychological factors at play.

This theoretical framework is particularly well‐suited for investigating uncivil patient behaviors (UPBs) among emergency department nurses. First, triadic reciprocal determinism offers an ideal lens for understanding how UPBs emerge from the interplay of the emergency department environment (environmental factor), nurses’ cognitive appraisals (personal factor), and their actual behavioral choices (behavior). Second, in contrast to the theory of planned behavior, which focuses primarily on behavioral intentions, SCT accounts for the dynamic, bidirectional relationships between context and cognition—a feature that is especially pertinent in emergency settings, where decisions must be made under extreme time constraints. Therefore, we adopted SCT as a guiding framework and employed a descriptive qualitative research design to explore the subjective life experiences of emergency department nurses regarding UPBs in China and aimed to systematically reveal the multidimensional sources, profound impacts, and complex coping mechanisms of such behavior. This study thus advances UPB theory by uncovering its processual dynamics and contextual contingencies in a previously underexplored high‐stakes healthcare setting.

## 2. Methods

### 2.1. Study Design

This study employed a descriptive qualitative approach and conducted semistructured interviews to examine the sources, impacts, and coping strategies of emergency nurses’ UPBs. Qualitative descriptive research is particularly suitable for exploring the lived experiences of specific groups in particular contexts while maintaining fidelity to the original data. This study’ *s* report follows the Consolidated Criteria for Reporting Qualitative Research (COREQ) [23].

### 2.2. Settings and Participants

Using purposive sampling, participants were recruited from five Grade II‐A or above hospitals in Zibo City, Shandong Province, China—namely, Zibo Central Hospital, Zibo Maternal and Child Health Hospital, Zibo Hospital of Traditional Chinese Medicine, Zichuan District Hospital, and Linzi District People’s Hospital.

Potential participants were recruited according to the following inclusion criteria: (a) were aged 18 years or older, (b) held a valid nursing practice certificate registered in an emergency department, (c) had a minimum of one year of work experience in an emergency department, and (d) provided written informed consent. Those involved in research management roles, without direct interaction with patients were excluded. Given the onus was on potential participants to indicate their interest in participating, reasons for nonparticipation are not known. Face‐to‐face interviews were conducted until data saturation was achieved, defined as the point at which no new themes or topics emerged.

### 2.3. Pilot Study

Prior to formal data collection, a pilot study was conducted to assess the clarity, relevance, and comprehensiveness of the semistructured interview guide, to test the feasibility of the recruitment and interview procedures, and to familiarize the interviewer with the interview process. Three emergency nurses who met the same inclusion criteria as the main study participants were purposively recruited to participate in the pilot interviews. These participants were not included in the main study sample.

### 2.4. Data Collection

Based on consultations with experts in qualitative research and clinical nursing practice, this study integrated research objectives with relevant literature and drafted an interview outline that operationalized SCT’ *s* triadic reciprocal determinism, with questions designed to capture organizational environmental factors, personal cognitive appraisals, and behavioral responses. The formal interview outline: (1) How do you perceive the “work rhythm” and “team collaboration model” in ED? (2) What do you consider the ethical climate like in emergency department? (3) Have you experienced situations where you perceived a scenario as ethically inappropriate but ultimately acted from an organizational perspective? Could you describe a specific scenario? (4) How do you view UPBs in the workplace? What factors do you believe contribute to their occurrence, and what impacts have they had on you? (5) How did you personally respond when such behaviors occurred, and how were they addressed by the organization and colleagues upon discovery? (6) Do you have any additional thoughts that need to be supplemented? Guided by the interview outline and employing exploratory questioning techniques, the researchers encouraged participants to express their perspectives, with interview sequences and content adjusted as needed during sessions. To ensure data integrity, two researchers jointly reviewed recordings within 24 h postinterview and transcribed audio content verbatim into written transcripts. The interview duration ranged from 22 to 53 min (mean = 32 min).

Researchers scheduled interviews in advance to maintain a structured schedule and minimize external interference in a controlled environment (interview room), ensuring rigorous data collection procedures. No one other than the participant or interviewer was present during the interview. The interviewer was a female master’s degree holder in nursing, systematically trained in qualitative research methods, and possessed over five years of clinical experience. With participants’ consent, the entire interview content was recorded in Chinese via voice recorder. Prior to each interview, participants were provided with a brief operational definition of UPB to establish a shared understanding of the construct. During interviews, the interviewer prioritized active listening while avoiding interruptions to participants’ narratives. Through timely summaries, in depth follow‐ups, and focused probes to verify that the discussed behaviors aligned with the UPB definition, and the interviewer effectively facilitated substantive dialog, ensuring comprehensive and accurate information collection. No prior relationship with participants existed. To confirm information saturation, an additional two interviews were conducted, after which no new themes emerged. Through iterative team discussions, we determined that the existing interviews provided sufficient informational depth to fully explore the study’s objectives. A total of 21 participants were included in the final analysis.

### 2.5. Data Analysis

The entire analytical procedure was functionally guided by the core tenet of SCT, namely, the triadic reciprocal determinism of organizational environmental factors, personal cognitive factors, and behavioral responses. The audio recordings were transcribed within 24 h, and the core members of the research team have a systematic background in qualitative research methodology; this study employs the inductive thematic analysis method for data analysis [24]. The researchers repeatedly listened to the recordings and studied the transcribed text. Initial codes were generated directly from participants’ accounts. Codes were grouped into potential themes, which were iteratively reviewed, refined, and defined to ensure accurate reflection of the dataset, with representative quotes selected to illustrate the themes. To ensure objectivity, data analysis was initially undertaken by two researchers independently. Divergent interpretations and disagreements were discussed, refined, and resolved using a third researcher where necessary. The provisional findings were then shared with the wider research team for review, with findings further refined until final consensus was reached.

### 2.6. Rigor and Trustworthiness

Several strategies were adopted to ensure the credibility and trustworthiness of this qualitative study. Nurses with diverse demographic characteristics (age, education, professional title, and working years) were recruited to enrich data perspectives and information saturation. Preinterviews were conducted to refine the interview outline and establish researcher–participant trust before formal data collection. All formal interviews were conducted in reserved quiet rooms at participants’ convenient time. Researchers clarified the study purpose, anonymity, and confidentiality principles prior to each interview to facilitate authentic participant expression. A researcher maintained continuous communication with the department to sustain a natural research atmosphere throughout the investigation. The interviewer was trained and experienced in qualitative research. During interviews, the interviewer remained neutral, primarily listened to participants without subjective induction, and voice recordings to ensure complete and original data. Study documentation was managed by one researcher (J. Qiao), with all named authors able to access it for reference. Strict member checking was performed to verify data accuracy and analytical credibility. After transcript sorting and preliminary thematic analysis, we returned both verified transcripts and extracted thematic interpretations to all participants. Participants confirmed the consistency of transcript content with their original statements and validated whether the thematic findings reflected their real experiences. All participant feedback was carefully reviewed and incorporated to correct analytical bias. Two researchers independently completed full manual coding. The researchers cross‐checked coding results and resolved discrepancies through group discussion. Reflective memos were recorded throughout the analytical process to maintain reflexivity and minimize subjective interpretation, further ensuring the rigor of research findings.

### 2.7. Ethical Considerations

This study was approved by the Medical Ethics Committee of Zibo Central Hospital (Ethics Number: 2026023). The research was conducted in strict compliance with the principles of the Declaration of Helsinki. Nurses were invited to participate voluntarily, and written informed consent was obtained from all participants prior to the interviews. Participants could refuse to answer any questions or withdraw from the study at any time. The anonymity of the participants was ensured by deidentifying the interview transcripts so that the nurse being interviewed is denoted by “N,” i.e., N1 refers to Nurse 1. We also encrypted the computers and associated data so that only study members could access the data.

## 3. Findings

### 3.1. Participants’ Characteristics

A total of 21 emergency nurses participated in this study, including 18 females (85.7%) and 3 males (14.3%). Their mean age was 33.52 years (SD = 7.61, range 23–49 years), and their mean clinical experience in the emergency department was 9.76 years (SD = 7.92, range 2–32 years). Among the participants, 10 (47.6%) were emergency department specialist nurses, while the remaining 11 (52.4%) were general emergency nurses. The respondents were recruited from 5 different hospitals, including 3 Grade III hospitals and 2 Grade II hospitals. Detailed demographic characteristics are presented in Table 1.

**TABLE 1:** Demographic and professional characteristics of participants (*n* = 21).

Characteristic variable		Value	Characteristic variable		Value
Gender (*n*)	Female	18	Technical title (*n*)	Nurse	4
	Male	3		Primary nurse	5
Age (years)	Range	23–49		Nurse‐in‐charge	8
	Mean	33.52		Co‐Chief Superintendent Nurse	4

Experience in emergency department (years)	Range	2–32	Specialty type (*n*)	General emergency nurse	11
	Mean	9.76		Emergency specialist nurse	10

Education level (*n*)	Junior College	2	Night shifts per month	Range	0–10
	Undergraduate	17		Mean	4.57
	Postgraduate	2	Hospital level (*n*)	Grade II	5

Marital status (*n*)	Married	21		Grade III	16

### 3.2. Themes, Subthemes, and Codes

During the data analysis process, codes were developed and categorized into three themes and eight subthemes, which reflect the sources, impacts, and coping strategies of emergency nurses’ UPBs. Table 2 shows the themes and subthemes.

**TABLE 2:** The main themes and subthemes.

Themes	Sub‐themes
Sources of unethical pro‐organizational behavior	High‐pressure ambiguous work environment
	Protecting the department*’*s reputation
	Unethical exemplars

Impacts of unethical pro‐organizational behavior	Individual moral distress
	Normalization of organizationally protective compromises
	Coping strategies for unethical pro‐organizational behavior

Coping strategies for unethical pro‐organizational behavior	Conflict between ethical perceptions and decision‐making
	Adapting and staying silent to fit in

#### 3.2.1. Sources of UPB

The unethical behaviors observed in emergency department nurses are often not isolated incidents but rather the result of the interplay of organizational environment, group pressure, and individual cognition. Against this backdrop, the study identifies the following three core subtopics.

##### 3.2.1.1. High‐Pressure Ambiguous Work Environment

Under high‐pressure emergency resuscitation, nurses’ attention narrows to focus exclusively on the most urgent and specific task objectives (e.g., “maintaining vital signs”), entering a “cognitive tunnel.”

N6: “When there is a sudden surge in patient volume, priority must be given to critical tasks rather than addressing less urgent matters, as saving lives takes precedence.”

At this stage, participants commonly reported that broad ethical principles (such as respecting autonomy and providing comprehensive informed consent) were perceived as “non‐urgent” and were temporarily deprioritized.

N7: “Emergency work is fast‐paced and detail‐oriented, requiring intuition and experience to determine the most urgent issues at hand.”

Continuous overwork, sudden emergency resuscitation tasks, and ambiguous ethical decision‐making boundaries collectively create fertile ground for UPBs, leaving emergency department nurses often feeling “with no other options.”

N11: “The resuscitation room often has endless shifts, with many night shifts and immense pressure. Once on duty, it’s like operating at full throttle. The ideal scenario—addressing every task and caring for every patient to perfection—is almost impossible… I have no choice.”

##### 3.2.1.2. Protecting the Department’s Reputation

Some nurses may conflate compliance with departmental “unspoken rules” or management’s singular focus on efficiency with organizational loyalty, thereby overriding personal ethical judgment. Under such cognitive bias, fundamental ethical principles are eclipsed by the imperative of “organizational excellence,” leading nurses to unconsciously deviate from professional ethics.

N1: “Omissions in nursing documentation directly impact departmental evaluations. Thus, colleagues modify documentation entries to make records appear more “perfect.” This serves collective departmental honor and does not constitute fraud.”

When improper practices occur in the department, nurses often opt for silence, concealment, or nonreporting, equating “protecting the department’s reputation” with loyalty to the organization, thereby suppressing the ethical requirement of honesty and transparency.

N7: “Once, a doctor instructed me to administer medication after the patient had finished vomiting. However, the patient appeared intensely nauseated and distressed, so I made the judgment call to administer it earlier to relieve the patient’s suffering. I later communicated this to the doctor, who agreed with my decision. The patient experienced no adverse effects. However, I still worried—what if the patient or family misunderstood my intent and filed a complaint against the department? I hesitated to speak up about this concern and chose to keep silent for the sake of the collective.”

##### 3.2.1.3. Unethical Exemplars

Senior colleagues or managers’ “shortcut” behaviors during busy periods (such as aggressive communication or differential treatment) exert a significant negative modeling influence on new nurses, fostering a perception among them that these actions are “acceptable norms.”

N11: “When Teacher Wang was repeatedly questioned by family members last time, she responded: I suspect acute abdomen now. We need test results first. You won’t understand if I explain too much. Pay for tests and get checked immediately to avoid delays! The family members immediately calmed down. Sometimes ‘straightforward approaches’ prove more effective.”

Organizational culture and ethical climate are critical determinants influencing nurses’ willingness to report observed unethical behaviors (whistle‐blowing). When departments develop unspoken rules that conceal misconduct under the guise of “unity,” a culture lacking accountability and integrity suppresses whistleblowing, transforming individual silence into a collective norm of tacit acceptance.

N2: “I don’t find it unusual. We even discuss it privately. The head nurse hasn’t punished anyone, but reminds us to be more careful next time. If one insists on pursuing ethical paths in isolation, one will not endure within the team. After all, we all work together and must adapt to the team’s rhythm and work philosophy.”

#### 3.2.2. Impacts of UPB

UPBs present a paradoxical challenge for emergency department nurses. While they may appear to protect departmental interests in the short term, they can undermine nurses’ mental health, precipitate moral dilemmas, accelerate occupational burnout, and erode the ethical cultural foundation of the organization over the medium to long term.

##### 3.2.2.1. Individual Moral Distress

Nurses who engage in or witness UPBs often experience profound inner conflicts and moral distress. This distress stems from the disconnection between professional ethical awareness and actual actions: While nurses are aware that certain behaviors violate ethical principles, they are compelled to act or remain silent due to organizational loyalty or practical pressures. The interviewees’ accounts authentically reflect feelings of guilt, indecision, and powerlessness.

N15: “I inserted a urinary catheter without providing thorough education to a 17‐year‐old girl. After the procedure, she cried. I assumed it was pain, but she said she could not tolerate the lithotomy position… I should have been more careful… This incident has haunted me for years. While it improved work efficiency, it left a psychological scar on her [tears in the nurse’s eyes]. I was overwhelmed at the time and did not intentionally neglect her needs. Looking back, I still deeply regret it.”

The impact of moral distress on nurses is multidimensional and profound. Repeated exposure to ethical dilemmas serves as a significant source of occupational burnout and professional identity erosion. Such distress may manifest as silence or social withdrawal, hindering open communication and collaborative care essential for patient safety.

N1: “ED work involves countless scenarios—such as administering the wrong medication (same drug, just incorrect bed number). Progressing from initial guilt to fear of potential complications, and eventually to burnout over time, I’ve come to accept these situations as routine while constantly questioning: Is this truly my profession’s expectation? These emotions resonate deeply, especially during quiet moments of reflection… My thoughts become tangled with complex emotions (nurse falls into deep contemplation).”

##### 3.2.2.2. Normalization of Organizationally Protective Compromises

An organizational‐level impact of UPBs described by participants was the normalization of organizationally protective compromises. When UPBs persist without effective intervention, practices that prioritize departmental reputation, conflict avoidance, or performance indicators over patient‐centered ethical considerations may gradually be accepted as “part of the job.” In this study, normalization does not refer to ordinary clinical coping under resource scarcity; rather, it refers to the repeated and socially shared acceptance of such compromises, even when participants recognized them as ethically problematic.

N18: “In the ER, we ban conscious patients from using phones. We cite equipment interference, but the real reason? We’re afraid they’ll film us—capture a mistake and put it online. Some patients feel utterly isolated because of this rule, even when they protest. From a team perspective, workplace safety takes precedence over everything else. Excessive empathy would undermine professional responsibility!”

When such compromises are tacitly tolerated and normalized, safe and open communication is suppressed, monitoring mechanisms (such as reporting systems) becomes ineffective, and “organizational compliance” takes precedence over “patient care.” Nurses’ professional decision‐making becomes distorted.

N19: “During shortages of emergency equipment (e.g., monitors and ventilators), we sometimes prioritize allocating devices to patients with ‘higher treatment potential’ or ‘lower dispute risks.’ This strategy improves ‘rescue success rates’ (departmental data) while reducing conflicts (some critically ill patients’ families may refuse equipment use due to communication barriers, and mandatory implementation could trigger complaints). Such scenarios are frequently observed.”

#### 3.2.3. Coping Strategies for UPB

Nurses’ responses are not static but rather dynamic, oscillating between the “ought” of professional conscience and the “ought‐to‐be” imposed by departmental demands. Within this oppressive and high‐pressure system, direct individual resistance often incurs prohibitive costs, leading nurses to construct “psychological safety islands” and form tacit alliances with trusted colleagues.

##### 3.2.3.1. Conflict Between Ethical Perceptions and Decision‐Making

The emergency department environment is unique, requiring decisions under time pressure and limited resources. Emergency department nurses frequently face ethical dilemmas between saving lives and patient autonomy, truth‐telling and protection, fairness and utility, and team directives and personal judgment. This “perceived conflict” itself reflects a positive ethical awareness within the profession.

N10: “When someone dies at home, families often demand transport to the Emergency Room—they believe dying at home brings bad luck. So doctors give in, and we work the code all the way to the hospital. The patient wouldn’t have wanted this, but we can’t say no. It’s not just about obligation—it’s about preventing complaints. Ethical practice? Sometimes it feels impossible.”

##### 3.2.3.2. Adapting and Staying Silent to Fit in

In ethical dilemmas, some nurses—particularly those with limited experience—often tend to observe and emulate colleagues’ common practices or seek allies to “adapt” to the environment, gain recognition, or alleviate psychological stress.

N17: “I once saw a colleague keep unused meds instead of returning them to patients, then use them elsewhere. I wanted to say it wasn’t right, but another nurse warned me: ‘If you speak up, everyone will think you’re the one who oversteps and sticks to principles too rigidly—it’ll hurt teamwork.’ Eventually, I stopped caring‐I just needed to fit in. Besides, the supervisor never really punished anyone for it.”

In emergency department settings, some nurses—particularly those with limited experience—often tend to adapt to the department’s “unified rhythm” or remain silent when ethical concerns arise, in order to maintain efficient operations and avoid disrupting the team.

N3: “Emergency departments operate on team‐based systems requiring unified rhythms. Special requests (e.g., insisting on spending excessive time with patients deemed critical) immediately cause resource congestion and system breakdowns, rendering operations impossible. Adaptation or silence becomes the only choice.”

Adjusting one’s actions or attitudes to align with group norms becomes the most direct way to gain social recognition and a sense of belonging. Public criticism may be perceived as “undermining unity,” while compliance serves as a signal of loyalty and the pursuit of acceptance.

N19: “This is the survival rule in emergency departments. When ethical principles clash with departmental conventions, temporary hesitation is followed by compliance as the only option.”

## 4. Discussion

Understanding how UPBs manifest in high‐stakes, collectivistic healthcare settings is essential for developing ethically sustainable emergency care environments. Drawing on the experiences of 21 emergency nurses in a Chinese city, this descriptive qualitative study offers empirical insights into the nuanced, context‐specific manifestations of UPBs within the structural and emotional landscape of emergency nursing, thereby providing evidence to inform the construction of an ethically supportive nursing environment. Notably, the predominance of female participants (*n* = 18, 85.7%) reflects the gender composition of the nursing profession in China and aligns with previous nursing research conducted in similar contexts. The inclusion of nurses from both tertiary (Grade III) and secondary (Grade II‐A) hospitals offers a more comprehensive view of UPBs across different institutional resource levels, as nurses in better‐resourced tertiary hospitals may face different ethical pressures compared to those in secondary hospitals with greater resource constraints. Furthermore, participants with longer tenure (≥ 10 years) often provided richer narratives regarding the normalization of UPBs over time, suggesting that experience may shape both awareness of and adaptation to ethical compromises. Data collection and analysis continued until no new codes or themes emerged, and the research team agreed that thematic saturation had been achieved. While our sample size precludes statistical generalization, the demographic diversity within our sample strengthens the credibility and contextual relevance of our findings. Our analysis yielded three key findings: the sources of UPBs rooted in organizational dynamics, their multifaceted consequences, and the paradoxical coping strategies employed by nurses. The extreme stress and uncertainty inherent in emergency care foster such unethical conduct. Under conditions of “time urgency, incomplete information, and significant consequences,” efficiency becomes the paramount criterion for decision‐making and action. Under such efficiency‐driven logic, nurses’ behavioral intentions toward UPBs were primarily shaped by organizational imperatives and situational constraints rather than individual ethical considerations. This “firefighting mode” implicitly establishes standardized, seemingly “uncompromising” team rhythms as the ultimate operational principle, legitimizing unethical practices such as “prioritizing resuscitation over ethics” and “serving organizational interests.” Emergency department nurses continually grapple with the fundamental conflict between “doing the right thing” and “doing what is feasible,” a structural contradiction that directly fosters ethical dilemmas. This compels individuals to seek survival strategies through compromise, prioritizing organizational interests over ethical reflection, adopting “adaptive compliance” or “moral disengagement,” and gradually developing coping mechanisms in unethical scenarios. These findings underscore the need to examine macrolevel factors such as organizational support, ethical leadership, and institutional design to explore pathways for creating emergency care environments that support ethical practices. This study addresses the theoretical and practical imperative of understanding UPBs in high‐stakes, collectivistic emergency department contexts, providing evidence to inform context‐sensitive interventions.

This study set out to describe the sources, impacts, and coping strategies of UPBs among emergency department nurses. Our analysis identified three themes and eight subthemes. Taken together, participants’ accounts suggest that UPBs in this context may arise from the interplay of a high‐pressure ambiguous work environment, loyalty framed as protecting the department’s reputation, and unethical exemplars. These behaviors were described as producing individual moral distress and, over time, becoming normalized, while nurses’ coping strategies oscillated between ethical conflict and silent adaptation. Notably, these findings are descriptive in nature and constrained by the contextual boundaries of the present investigation.

Studies have shown that protecting the organization’s reputation is fundamentally about preserving the functional integrity of an overburdened unit in the emergency department context [25, 26]. Nurses who engage in UPBs appear driven not by self‐interest but by a distorted sense of altruism toward colleagues and patients. This motivation complicates the traditional view of UPBs as purely self‐serving behavior. Critically, this tendency toward “well‐intentioned” rule‐bending is not merely an individual phenomenon but is socially propagated through the organizational hierarchy. Our findings showed that unethical role modeling is a key source of UPBs transmission, highlighting the trickle‐down effect of destructive leadership in fast‐paced medical environments. When head nurses or senior physicians implicitly endorse “cutting corners” to achieve performance metrics, they inadvertently construct an ethical climate of expediency, which is exacerbated by the hierarchical culture of emergency department nurses, where questioning authority is often discouraged. While previous studies have shown that misconduct in healthcare can damage the reputation of the nursing profession and the organization [27, 28], our findings extend this understanding by identifying the origins of such misconduct in the systemic entanglement of organization’s reputation, leadership, and structural pressures.

The absence of ethical leadership and the dominance of efficiency‐oriented pragmatism create an “ethical vacuum” that enables UPBs proliferation. In the emergency department environment, cultivating an ethical climate faces unique challenges: Ethical reflection is marginalized, and ethical decision‐making is reduced to considerations of efficiency‐oriented pragmatism [29]. The lack of ethical leadership further exacerbates this dilemma [30]. Ethical leadership in nursing is crucial for fostering a transparent workplace culture and encouraging error reporting [31]. When leaders fail to proactively discuss ethical dilemmas, incorporate ethical considerations into resource allocation, or address unethical behavior rather than tolerate it, an “ethical vacuum” is created that enables the proliferation of UPBs. In the high‐power distance cultural context, subordinates may adopt a more tolerant attitude toward UPBs to obtain career development resources [32]. Nurses may perceive organizational indifference toward ethical issues, leading them to view UPBs as acceptable “adaptive strategies” and to develop an illusion of “compliance with organizational atmosphere.” Adequate organizational support, including human, equipment, and time resources, a positive ethical climate, and ethical education (ethical competence and support) can reduce nurses’ exposure to ethical dilemmas and minimize the need for UPBs [33]. These results suggest that in a collectivist cultural context, the ethical climate at the organizational level and behavioral modeling profoundly influence individual ethical decision‐making.

Cultural‐related workplace norms—particularly collectivist‐oriented group harmony expectations and local workplace concerns about “mianzi” (face)—interact with organizational support deficits to shape nurses’ situational ethical compromises. Within high‐pressure emergency department settings, nurses often experience insufficient organizational support amid heavy workloads, alongside workplace norms that prioritize group harmony and institutional interests in the sampled clinical context [34]. Consequently, nurses may be compelled to make ethical compromises under resource constraints, triggering a rationalization mechanism of “sacrificing ethical principles for organizational interests.” Furthermore, interpersonal face‐maintenance norms prevalent in local clinical settings appear to reinforce this tendency [16]. In day‐to‐day clinical practice, prioritizing relational considerations or responding to leadership expectations is sometimes perceived as a pragmatic way to sustain workplace interpersonal harmony. Our results suggest that UPBs may function as a context‐specific coping strategy, consistent with Lipsky’s theory of “street‐level bureaucracy” [35]. This combined influence of workplace relational logic and institutional environment appears to be associated with the subtle erosion of ethical principles during routine clinical operations. Additionally, emergency department nurses often face performance evaluation systems that overemphasize efficiency‐oriented indicators, such as resuscitation success rates and bed turnover rates, while paying less attention to ethical‐quality indicators, such as patient‐centered satisfaction and the quality of ethical practice [36]. This institutional orientation may send implicit workplace signals that operational efficiency and institutional goals take priority over individual ethical integrity, which participants perceived could generate situational pressure to compromise ethical standards in order to meet quantitative performance targets.

UPBs may be associated with dual‐level harm to individuals and organizations through moral distress and the normalization of such behaviors. Our findings indicate that, from participants’ accounts, UPBs are linked to adverse impacts on individual and organizational safety among emergency department nurses, primarily manifested as individual‐level moral distress and the normalization of UPBs. Nurses in this study reported experiencing exhaustion, anxiety, and low mood at physical and psychological levels. Participants further described sustained moral distress as a potential contributor to burnout, while the hospital’s ethical climate appeared to function as an intervening factor in the relationship between moral distress and burnout [36]. Widespread individual moral distress normalizes at the organizational level, leading to collective ethical alienation and systemic dysfunction. Reinforced by the intergenerational transmission of UPBs, the organization falls into collective stagnation, culminating in a toxic ethical environment within departments. The consequences transcend individual psychology; they signal a systemic failure of the ethical infrastructure, in which the organization becomes reliant on the moral compromises of its workforce to maintain operational functionality. Furthermore, negative cultural phenomena such as organizational bullying are significantly positively correlated with nurses’ ethical distress and moral disengagement [24], a positive ethical climate is significantly positively correlated with nurses’ job engagement, while the perception of ethical climate directly influences their levels of occupational burnout [37]. Therefore, enhancing nurses’ awareness of organizationally condoned unethical behaviors in emergency department nurses and fostering a positive ethical climate are urgent and essential tasks that require widespread attention from organizations, managers, and other stakeholders.

Moral distress, rather than signaling ethical failure, reflects nurses’ intact ethical awareness and points to systemic inadequacies that require organizational‐level remedies. The moral distress perceived by emergency department nurses serves as a signal of their unextinguished ethical conscience and a warning of the need for improvement in organizational ethical environments. As unethical behaviors normalize, nurses shift from moral distress to learned helplessness, rationalizing (e.g., “everyone does this”) and internalizing them (e.g., “departmental tradition”) to reduce cognitive dissonance [12]. This process may even be institutionalized as a so‐called “departmental experience” through intergenerational transmission [3]. These observations point away from individual accountability and toward the organizational ethical climate. Prior research suggests that several organizational measures may be effective in this regard. Baseline assessments of ethical climate have been found to help clarify how nurses’ moral distress arises [38]; continuous monitoring and evaluation have been reported to support regular review of implementation and ease nurses’ concerns about retaliation or negative consequences [39]. It seems reasonable to consider these measures in emergency department settings, while their effectiveness in this specific context requires further verification.

Ethical conflicts triggered by UPBs are managed through moral disengagement, yet this coping mechanism risks progressing from temporary rationalization to permanent moral numbing. Our findings showed that emergency department nurses experienced ethical perceptions and decision‐making conflicts when confronted with UPBs. In high‐pressure, uncertain work environments such as emergency department nurses, nurses are prone to ethical conflicts, often manifesting as the paradox of violating protocols to ensure organizational efficiency [1]. When facing ethical conflicts, nurses often resort to moral disengagement for self‐regulatory rationalization: “It’s all for the organization,” “I had no choice,” or “Others achieved better outcomes.” However, this decoupling signifies a critical erosion of moral resilience. Frequent rationalization may gradually blunt ethical sensitivity, turning “moral distress” into “moral numbness.” This intrapsychic process appeared to be socially reinforced, as participants used colleagues’ behavior as tacit cues for acceptable practice [40]. In our setting, “adaptive conformity” operated more as a situational coping strategy than as pure informational learning. However, our data suggest that once routinized, such conformity may facilitate UPBs’ transition from isolated deviations to collective norms and potentially into the ward’s organizational culture.

The progression from conflict to rationalization to compliance emerged in participants’ accounts as a dynamic continuum reflecting nurses’ passive adaptation to systemic pressures. Emergency nurses’ responses to UPBs were not described as static or singular, but as a dynamically evolving psychological‐behavioral process beginning with ethical perception and decision‐making conflict. At the organizational level, prior work on ethical leadership suggests that management should explicitly oppose unethical practices justified in the name of organizational interests and replace negative ethical exemplars with positive role models and transparent ethical decision‐making processes [30, 31]. At the support‐system level, existing studies support establishing regular ethical case discussions, accessible ethical consultation channels, and institutionalized ethical support mechanisms, such as practical ethics committees that shift from postevent adjudication to preevent consultation [41]. At the individual level, integrating the identification of moral distress into employee mental health support systems [42]—for example, specialized counseling for moral trauma and group counseling—may help nurses process ethical trauma and prevent apathetic rationalization. Finally, at the environmental and resource level, prior research indicates that optimizing environmental design, rationally allocating human resources, and encouraging interdisciplinary and cross‐level ethical dialogs can enhance staff ethical literacy [43, 44].

## 5. Conclusions

This study described the behaviors, influences, and consequences of nurses’ UPBs as reported by participants. UPBs were portrayed as offering short‐term benefit while carrying severe long‐term harms, suggesting that nurses’ awareness of these consequences may warrant attention. Future research and management could explore areas such as quality control, institutional standardization, education and training, and multifaceted support. These findings may contribute to understanding UPBs in nursing, offer a descriptive basis for further research, and extend discussion of nursing ethics and UPBs.

## 6. Limitations

This study has some limitations. First, it was conducted in five hospitals in Zibo, Shandong Province, which may limit the transferability of our findings to emergency nurses practicing in other geographical or cultural settings within China. Future research should consider multicenter designs across diverse regions to validate and extend our results. Second, the sensitive topic may invite social desirability bias, for which we employed anonymization and confidentiality assurances to mitigate. Consistency of narratives across participants suggests that core patterns are unlikely to be artifacts of response bias alone. Third, the predominantly female sample, while reflective of the nursing workforce, limited exploration of potential sex‐related differences in ethical reasoning and UPBs rationalization. Collectively, these limitations position this study as an initial exploratory step; therefore, future studies should include broader geographic, demographic, and methodological scopes and employ multicenter designs, balanced sex samples, and observational or longitudinal approaches to illuminate the systemic dynamics of UPBs in emergency nursing.

## Author Contributions

Juan Qiao: writing–review and editing, writing–original draft, methodology, investigation, formal analysis, data curation, and conceptualization. Junjie Ma: writing–review and editing, project administration, formal analysis, and conceptualization. Huiling Feng: supervision, data curation, and conceptualization. Shanshan Zhang: resources, methodology, formal analysis, and data curation. Lin Wang: supervision, data curation, and conceptualization. Minghui Zhou: writing–review and editing, supervision, resources, and methodology. Hanfei Cui and Xuebing Jing: writing–review and editing, supervision, resources, methodology, formal analysis, and conceptualization.

## Funding

This research did not receive any specific grant from funding agencies in the public, commercial, or not‐for‐profit sectors.

## Ethics Statement

This study was approved by the Medical Ethics Committee of Zibo Central Hospital (Ethics Number: 2026023).

## Conflicts of Interest

The authors declare no conflicts of interest.

## Data Availability

Data extraction sheets and further information are available from the corresponding authors on request.
